# Metastatic Adenocarcinoma of the Prostate to the Brain Initially Suspected as Meningioma by Magnetic Resonance Imaging

**DOI:** 10.7759/cureus.12285

**Published:** 2020-12-25

**Authors:** Young Son, Paul Chialastri, Julia T Scali, Thomas J Mueller

**Affiliations:** 1 Urology, Rowan University School of Osteopathic Medicine, Stratford, USA; 2 Urology, New Jersey Urology, LLC, Voorhees, USA

**Keywords:** prostate cancer, brain metastasis, craniotomy, meningioma, prostatic adenocarcinoma, prostatic metastasis

## Abstract

Brain metastasis from prostate cancer is rare, occurring in less than 1% of metastatic prostate cancer patients. Brain metastasis can cause edema, neurologic symptoms, and may be misdiagnosed as primary brain tumors on imaging. A 68-year-old male presented to the emergency department complaining of headaches, right-sided weakness, multiple falls, and a 45 pounds of unintentional weight loss. Computerized tomography (CT) scan without contrast of the head showed a 3.2 cm right frontal mass with edema suspicious for meningioma. Associated nonspecific bony lesions were found on CT of the abdomen and pelvis. Magnetic resonance imaging (MRI) of the brain showed a 2.8 cm right frontal mass with an enhanced dural tail. Preoperative labs were noteworthy for a hemoglobin of 9.7 and prostate-specific antigen (PSA) of 66.7 ng/ml. Craniotomy with resection of tumor was performed with a frozen sample diagnosed as meningioma. Permanent pathology with stains were positive for PSA and prostatic-specific acid phosphatase (PSAP), making the diagnosis of metastatic prostate adenocarcinoma. Postoperatively, nuclear bone scan showed uptake in the axial skeleton consistent with metastasis. After the diagnosis of metastatic prostate cancer was made, bicalutamide was administered followed by degarelix with plans to transition to leuprorelin one month later. This is to be followed up by whole brain radiation therapy (WBRT). PSA was 118.53 ng/ml three weeks after craniotomy, but prior to androgen deprivation therapy.

Metastatic prostate cancer can present with neurological symptoms most commonly following spread to the axial skeleton and impingement of the spinal cord. Metastasis to the brain is rare and is usually associated with vague symptomatology depending on extent and location of the lesion. While brain metastasis can occur in known prostate cancer patients, this case shows that metastasis can occur prior to any formal prostate cancer diagnosis and can be mistaken for meningioma on imaging and frozen sectioning. Practitioners must be vigilant, and precautions should be taken to rule in metastatic prostate cancer as a possible cause for a brain lesion in patients of the appropriate demographics.

## Introduction

Prostate cancer is the second-leading cause of cancer death in men after lung cancer. The most common site of prostate metastasis is bone (84%), lymph node (10.6%), liver (10.2%), and thorax (9.1%) with 18.4% to multiple metastatic sites [1]. Prostate metastasis to the brain is rare with less than 1% documented cases from MD Anderson Cancer Center [2]. It is estimated that 1%-6% of prostate cancer metastasis is found in post-mortem examination [3]. Parenchymal brain metastasis has a mean survival of 9.2 months after discovery of brain metastasis [4]. Acute neurological symptoms of metastatic prostate cancer are observed with metastasis to the lumbar spine that impinges the spinal cord due to bony lesions. Symptoms of spinal cord compression, urinary retention, and hematuria are commonly seen. Conversely, meningioma is a slow growing, non-infiltrative tumor that commonly presents with seizures, headaches, and focal neurologic deficits depending on location [5]. Prostate cancer is often diagnosed with neurological symptoms along with increasing prostate-specific antigen (PSA). Metastatic prostate cancer presenting with headaches with normal levels of PSA is a rare presentation. We report a 68-year-old male that presented to the emergency department with a primary complaint of headache and falls that were initially diagnosed as meningioma with magnetic resonance imaging (MRI) and intraoperative frozen section that was later confirmed to be metastatic adenocarcinoma of the prostate on pathology.

## Case presentation

**Figure 1:**
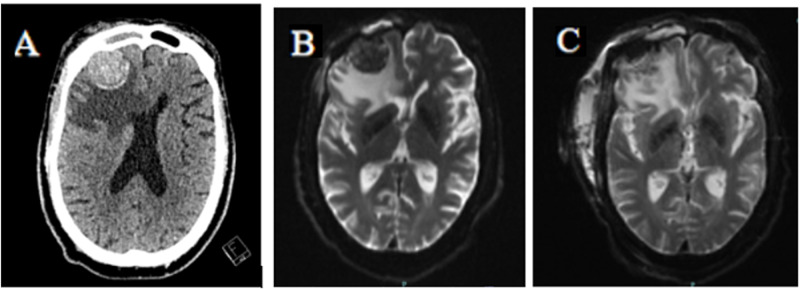
(A) Preoperative transverse CT. (B) Preoperative transverse MRI. (C) Postoperative transverse MRI. (A) Preoperative CT head showing 3.2 cm right anterior frontal mass with edema. (B) Transverse view of preoperative MRI showing 2.8 cm right anterior frontal extra-axial mass with enhancing dural tail, considered to likely be a meningioma. Moderate amount of vasogenic edema was considered unusual and raised considerations for other histology such as oligodendroglioma by radiology. (C) Postoperative day two MRI showing post-surgical changes and complete removal of mass. CT, Computed tomography; MRI, magnetic resonance imaging.

**Figure 2:**
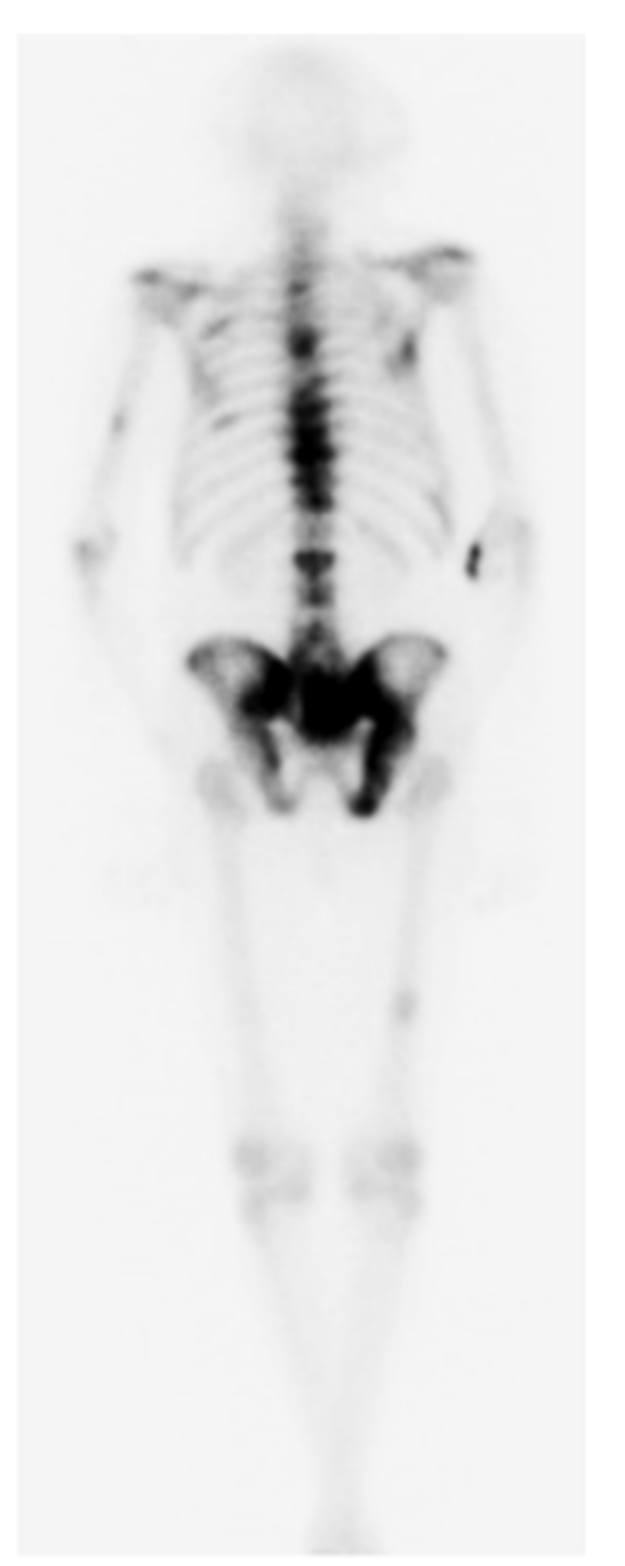
Posterior view bone scan Posterior view of bone scan showing increased uptake in thoracic and lumbar vertebral bodies, sacrum, left humerus, right femur, and bilateral ribs.

A 68-year-old male presented to the emergency department with headaches, right-sided weakness, and multiple falls for one-month duration. He was noted to have had 45 pounds of unintentional weight loss over the four months prior to presentation. Initial CT of the head showed a 3.2 cm right frontal mass with edema that was suspicious for meningioma, and the patient was admitted for further investigation and treatment. A metastatic workup was delayed by the urgent craniotomy due to mass effect at presentation. Preoperative workup showed anemia with hemoglobin of 9.7 g/dl, alkaline phosphatase of 144 IU/L, and elevated PSA of 66.7 ng/ml. Digital rectal exam revealed asymmetry of the prostate with an elevation of the left side of the gland but no discrete nodules. MRI of the brain showed a 2.8 cm right anterior frontal extra-axial mass with enhancing dural tail, likely representing meningioma. Additionally, MRI showed moderate vasogenic edema, raising suspicion for other histology such as oligodendroglioma (Figure 1). Craniotomy was performed with an intraoperative frozen section initially reported as meningioma but subsequently diagnosed as metastatic adenocarcinoma of the prostate on permanent pathology. Bone scan postoperatively showed increased uptake in multiple thoracic and lumbar vertebral bodies, sacrum, left humerus, right femur, and several bilateral ribs, suspicious for metastatic disease (Figure 2). There was also an increased uptake in the right anterior skull, suggestive of post-surgical change. The CT abdomen and pelvis showed patchy sclerosis throughout the thoracolumbar spine and bony pelvis, suspicious for osteoblastic metastasis. Three weeks later, the patient presented to the hospital due to lower extremity edema. At this time, repeat PSA was found to be 118.53 with total testosterone of 144 ng/dL. During the hospital stay, bicalutamide was initiated and then was followed by degarelix in the clinic one week later. Leuprorelin was subsequently planned for one-month post operatively and followed up with whole brain radiation therapy (WBRT).

## Discussion

Differentiating primary brain lesions from metastatic prostate cancer can be challenging, especially when a solitary lesion is present. Specific MRI characteristics from metastatic prostate cancer have not been well established in literature; however, some reports describe hemorrhagic brain metastasis, mixed cystic, solid, or ring-like appearances on MRI. Hemorrhagic brain metastasis is more consistent with renal cell carcinoma, melanoma, choriocarcinoma, breast cancer, or thyroid cancer [6]. Of note, our patient had a negative head CT in December 2018. The rapid growth of the brain lesion also favors metastasis instead of meningioma as the cause. For our patient, the differential diagnosis of meningioma with edema was initially explored and then falsely positive confirmed with a frozen tissue sample biopsy. The initial sample did stain for meningioma.

Our patient’s PSA of 66.7 ng/ml qualifies for a bone scan for metastasis; however, the patient’s indolent course of prostate cancer was only detected at the time of extensive metastasis without causing lower urinary tract symptoms or other neurological symptoms usually seen with metastatic prostate cancer. Additionally, the patient’s previous PSA levels of 1.14 ng/ml in 2008, 0.91 ng/ml in 2011, and 1.18 ng/ml in 2013 along with the absence of symptoms showed no indication for a bone scan. This patient's intraoperative frozen pathology was reported as meningioma but later was documented as “cannot rule out malignancy.” Permanent pathology stained positive for PSA and prostatic-specific acid phosphatase (PSAP), supporting metastatic adenocarcinoma of prostatic origin as the diagnosis.

Brain biopsy is not recommended in metastatic prostatic disease. Although histopathology is the confirmatory test of choice, it is unnecessary in patients with poor prognoses. In our patient, the diagnosis of brain metastasis was confirmed post resection of brain tumor. With headache as the presenting problem, a primary brain tumor was more probable than metastatic disease. Routine head imaging is also not performed in patients with prostate cancer. Historically, it has been reported that solitary brain metastasis has been treated with craniotomy with resection followed by radiation therapy with possible WBRT [7]. Androgen deprivation therapy with bicalutamide and degarelix was initiated with systemic chemotherapy to follow. Currently, enzalutamide is the most commonly prescribed compound for treatment of metastatic castration-resistant prostate cancer. However, its use may be contraindicated in patients with brain metastasis since it potentiates seizures by crossing the blood-brain barrier. There are other antiandrogens, such as abiraterone and apalutimide, which are approved for use in metastatic prostate cancer, but with less passage through the blood-brain barrier, theorizing their limited use in brain metastasis [8]. Cabazetaxel has shown to improve passage through the blood-brain barrier compared to docetaxel [9]. Chemotherapy will be followed up by WBRT recommended by radiology oncology.

## Conclusions

Metastatic prostate cancer presenting as headaches due to metastasis to the brain is a rare finding that can occur at relatively low PSA levels. In patients of appropriate demographics and with abnormal digital rectal exams, differential diagnosis of prostate cancer must be explored even with imaging or histopathology that does not represent metastatic disease.
